# Upper airway dynamics during negative expiratory pressure in apneic and non-apneic awake snorers

**DOI:** 10.1186/1465-9921-7-54

**Published:** 2006-03-30

**Authors:** A Ferretti, P Giampiccolo, S Redolfi, S Mondini, F Cirignotta, A Cavalli, C Tantucci

**Affiliations:** 1 Division of Pneumology, S. Orsola-Malpighi Hospital, Bologna, Italy; 2 Pneumology Unit, ASL, Imola, Italy; 3 Respiratory Medicine Unit, Department of Internal Medicine, University of Brescia, Italy; 4 Neurology Unit, S. Orsola-Malpighi Hospital, University of Bologna, Italy

## Abstract

**Background:**

The ability of negative expiratory pressure (NEP) technique to differentiate between awake snorers with and without obstructive sleep apnea-hypopnea (OSAH) was investigated.

**Methods:**

Forty-eight subjects with sleep disordered breathing (SDB) and 7 healthy subjects, as non-snorer controls, underwent the NEP application of -5 and -7 cmH_2_O in the seated and supine position during wakefulness, after performing a sleep study. The upper airway collapsibility was assessed by computing the volume exhaled during the first 0.5 sec. (V,NEP_0.5_) and 1 sec. (V,NEP_1_) following the NEP start.

**Results:**

Patients with severe (AHI ≥ 30) (n = 19) and mild-to-moderate (AHI <30 and >5) (n = 15) OSAH had lower V,NEP_0.5 _(340 ± 88 ml) as compared to snorers (AHI ≤ 5) (n = 14) (427 ± 101 ml; p < 0.01) and controls (n = 7) (492 ± 69 ml; p < 0.001) in the supine position with NEP -5 cmH_2_O. Less significant differences among the different groups were observed for V,NEP_0.5 _in the seated position with NEP -5 cmH_2_O and in both positions with NEP -7 cmH_2_O (only OSAH patients vs controls, p < 0.001). Similar results were obtained for V,NEP_1 _in either position by using both NEP -5 cmH_2_O and -7 cmH_2_O. In spite of this, a substantial overlapping of V,NEP_0.5 _and V,NEP_1 _between snorers and OSAH patients did not allow to identify a reliable diagnostic cut-off level. An inverse correlation with AHI was found for V,NEP_0.5 _in the supine position with NEP -5 cmH_2_O (r_s _= -0.46, p < 0.05) in severe OSAH patients.

**Conclusion:**

The awake OSAH patients exhibit values of V,NEP_0.5 _and V,NEP_1 _lesser than those of awake snorers. The NEP technique, however, appears to have a limited usefulness as clinical tool for routine screening of the OSAH patients during wakefulness.

## Introduction

Among the mechanical factors that are believed to promote obstructive sleep apnea/hypopnea (OSAH), the increase in passive upper airway compliance, as assessed by the pharyngeal volume (area)-pressure relationship in the absence of upper airway dilator muscle activity, has been repeatedly emphasized [1-6]. This feature influences for a given transmural pressure the end-expiratory cross-sectional area at different levels of the upper airways and may be crucial for the development of upper airway narrowing and/or closure at the onset of inspiration during sleep, when the neural activation of upper airway dilator muscles decreases [7,8]. Moreover, the patients suffering from OSAH exhibited less negative (sometimes positive) closing (or critical) pressure of the passive upper airways (i.e. the pressure inside the upper airways when they close), as compared to sex, age and body mass index matched snorers and normal subjects [3,9-11]. The increased critical pressure that is considered to reflect a high extraluminal pressure has been ascribed in apneic patients to structural abnormalities such as para-pharyngeal fat deposits in obesity and/or reduced cross-section of bony structures of the lower face in cranio-facial anomalies [12,13]. In fact, several observations suggest that either obesity or cranio-facial anomalies would act to increase the tissue pressure surrounding the pharyngeal airway, thus favoring OSAH by reducing the transmural pharyngeal pressure and making the upper airways easier to narrow for a given compliance. In addition, there is compelling evidence that the upper airways have a smaller lumen during wakefulness [8,13,14] and sleep [3] in OSAH patients, who show an increase in the upper airway resistance [15-18], often assuming an anterior-posterior configuration of their major axis with a prevalent lateral narrowing [8,19]. These factors tend to increase both the pharyngeal compliance, which is volume and shape dependent, and the closing pressure. Recently, pharyngeal airway length has been found to be greater in OSAH patients, possibly influencing its collapsibility [20,21].

Hence, several, concurrent, inter-related mechanisms (increased compliance, decreased transmural pressure, smaller size and greater length of the upper airways) might enhance the pharyngeal collapsibility in patients with OSAH.

Therefore, simple assessment of upper airway mechanics during wakefulness could identify OSAH subjects and select them for standard polysomnography. In normal awake subjects the application of small negative expiratory pressure (NEP) transients at the onset of resting expiration does not elicit reflex activity of the genioglossus nor changes in upper airway resistance per *se *[22,23]. Under these conditions, the flow dynamics at the beginning of the expiratory phase during NEP application are expected to reflect the mechanical behavior of the pharyngeal airway in a "quasi-passive" condition even during wakefulness. Accordingly, the aim of our study was i) to investigate if volume exhaled during early application of NEP at the onset of quiet expiration at rest was different in OSAH patients, snorers and normal subjects, suggesting different degrees of pharyngeal collapsibility among these groups and ii) if these differences could be used to distinguish non-apneic from apneic snorers.

## Methods

### Subjects

In a prospective, randomized study we investigated at the Division of Pneumology of the S. Orsola-Malpighi Hospital of Bologna the early expiratory flow dynamics after the application of a small (-5 to -7 cmH_2_O) negative pressure at the mouth in 48 awake male subjects coming from the Neurology Unit who had performed a polysomnographic study in the Sleep Center because of suspected sleep disordered breathing. We excluded those with obvious anatomical defects such as cranio-facial and/or severe otorino-laryngoiatric (ORL) abnormalities, or with neurological and endocrine diseases known to be causally associated with SDB. Subjects affected by cardiac and respiratory disorders capable of causing intra-thoracic tidal expiratory flow limitation (EFL) were also excluded, as well as obese subjects with tidal intra-thoracic EFL in either position. Subjects were not treated with drugs active on CNS or suffered from chronic alcoholism. Among the enrolled subjects 34 resulted affected by obstructive sleep apnea-hypopnea (OSAH) and 14 were snorers without OSAH (Sn). Seven male subjects, non-apneic, non-snorer, as assessed by nocturnal polysomnography, were recruited from the Hospital staff as controls. The study was approved by the local Ethics Committee and an informed consent was obtained from each subject.

### Study design

#### Sleep study

All subjects were examined at the Sleep Center performing an overnight polysomnographic study by recording the following parameters: nasal pressure (by nasal cannula), oral flow (by thermistor), abdominal and rib cage movements (by piezo-sensors), oxygen saturation and heart rate (by finger oxymeter), snoring (by microphone), body movements and body posture. Respiratory events were defined as obstructive apnea in the presence of nose and mouth airflow cessation for at least 10 sec with concomitant inspiratory efforts and as obstructive hypopnea in the presence of discernable inspiratory airflow reduction with inspiratory efforts accompanied by a decrease of >3% in oxygen saturation. The results were expressed as the number of apnea and hypopnea per hour of sleep (apnea-hypopnea index, AHI) [24]. The subjects were categorized according to AHI as non-apneic snorers (AHI ≤ 5) and snorers with mild-to-moderate (AHI <30 and >5) or severe (AHI ≥ 30) OSAH.

#### NEP testing

**Figure 1:**
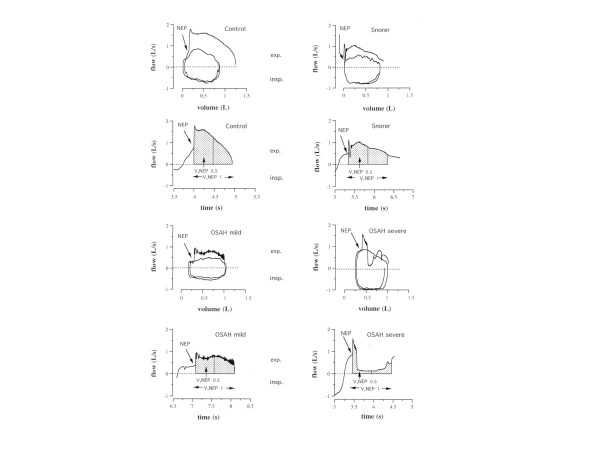
Supine tidal flow-volume curves (control and during NEP of -5 cmH_2_O) and  corresponding expiratory flow-time curves (only during NEP) in  representative subjects of the different groups. The hatched areas under the  flow measure the volume exhaled in the first 0.5 sec. (V,NEP_0.5_) and 1 sec.  (V,NEP_1_) after NEP application.

Subsequently, the subjects were sent to the Division of Pneumology to evaluate the upper airway mechanics looking at the flow-time relationship in the early tidal expiration during strict wakefulness. Expiratory flow dynamics was assessed during the application of a negative expiratory pressure at the mouth (NEP technique). NEP was applied randomly at two different levels, i.e. -5 cmH_2_O and -7 cmH_2_O, initially in the seated position and later, 10 minutes after assuming the supine posture. In both positions and at both levels of negative pressure, at least 5 NEP breath-tests were performed at intervals of 5–10 respiratory cycles, always when the patient had resumed regular breathing according to the spirogram that was continuously displayed on the computer monitor. In this respect, great care was placed to check the level of the end-expiratory lung volume. The expiratory flow recorded under each NEP application was measured in the first 0.5 and 1 sec from the onset of NEP administration to compute by time integration the volume exhaled in these time intervals, labeled hence fore V,NEP_0.5 _and V,NEP_1_, respectively (Fig. 1). For all subjects in each experimental condition (different posture and negative pressure levels) the mean value of V,NEP_0.5 _and V,NEP_1 _was calculated, after discarding the highest and the lowest value, by averaging those obtained during at least 3 acceptable NEP maneuvers. It should be noted that the NEP was applied unknown to the subject by a computer at the very onset of the tidal expiration. We also computed the differences between V,NEP_0.5 _and V,NEP_1 _and the corresponding volumes exhaled during preceding spontaneous expirations (ΔV,NEP_0.5 _and ΔV,NEP_1_) in the different groups of subjects. These measurements were performed in both positions and at the same different levels of NEP applied, aiming to normalize in each subject the V,NEP_0.5 _and V,NEP_1 _values for the baseline expiratory flows and volumes. The physician who performed and assessed the NEP tests was blinded to the polysomnographic results.

#### Pulmonary function testing

All subjects underwent spirometric measurements using a computerized system (Vmax 22; Sensor Medics, Yorba Linda, CA) in seated position. Slow vital capacity (VC) and three acceptable and reproducible maximal full flow/volume curves were obtained. Subjects inspired to TLC and then expired forcefully without an end-inspiratory pause to obtain forced vital capacity. The predicted values for volumes and flows were those proposed by the European Community for Coal and Steel [25].

### Experimental NEP set-up

In both seated and supine position, all subjects wearing nose-clips breathed spontaneously room air through a flanged mouthpiece and a heated pneumotachograph (3700 series; Hans Rudolph, Kansas City, MO) connected to a differential pressure transducer (Raytech DP55 ± 3 cmH_2_O; Raytech Instruments, Vancouver, BC, Canada) to measure the flow. The pneumotachograph was linear over the experimental flow range. Volume (V) was obtained by electrical time integration of the flow signal. Pressure was recorded at the mouth (Pm) via a rigid polyethylene catheter (internal diameter = 1.7 mm) connected to a differential pressure transducer (Raytech DP55 ± 100 cmH_2_O; Raytech Instruments). The pneumotachograph was assembled in series to a Venturi device that created a negative pressure in the circuit, whose magnitude could be precisely fixed. The application of the negative pressure did not affect the accuracy of the pneumotachograph which measured a flow less than 1 ml/s when the system was switched on. The Venturi device was connected to a solenoid valve (Asco electrical valve, model 8262G208; Ascoelectric, Ontario, Canada) controlled by a computer and automatically activated when the expiratory flow reached a pre-set threshold value (i.e.: 50 ml/s) and after a pre-set time delay (i.e.: 200 ms.) [26]. In all instances the NEP application was timed to last until the lung volume corresponding to the end-expiratory lung volume of the previous control breath was reached or for at least 1.3 sec. The flow and pressure signals were amplified (AC bridge Amplifier-ABC module; Raytech Instruments), filtered through a low-pass filter at 50 Hz, sent to an A/D converter (Direc Physiologic Recording System; Raytech Instruments) connected to an IBM personal computer and sampled at 200 Hz. Both digitized signals were displayed in real time on the computer screen together with the volume signal. The tracings were continuously monitored both with respect to time and as flow/volume curves. All signals were calibrated independently and simultaneously recorded on the hard disk of the computer and were used for subsequent analysis. Data analysis was performed using data analysis software (Direc NEP, version 3.1; Raytech Instruments or Anadat, version 5.2; RHT-InfoDat; Montreal, Quebec, Canada).

### Statistical analysis

Data are presented as mean ± standard deviation (SD). To assess and verify the normal distribution of the data in each group the Kolmogorov-Smirnov test was performed. Then, one-way ANOVA was used to compare data among groups both in seated and supine position and at different negative pressure and finally multiple comparisons, corrected by the Bonferroni method, were performed between groups, if allowed by the F-value. To assess differences in V,NEP_0.5 _and V,NEP_1 _within groups between seated and supine posture and different levels of negative pressure a paired Student's test was applied. Correlations between quantitative variables were performed using the Spearman's rank-order test. A p value less than 0.05 was considered statistically significant. The receiver-operating characteristic curves (ROC) were performed to assess sensitivity and specificity of V,NEP_0.5 _and V,NEP_1 _obtained with different levels of NEP in both positions to get optimal cut-offs.

## Results

The anthropometric and functional characteristics of the subjects are shown in Table 1. Snorers and OSAH patients were well matched, but patients with severe OSAH were older and had greater BMI than controls (p < 0.01). No correlation, however, was present between V,NEP_0.5 _and V,NEP_1 _and BMI in snorers and OSAH patients. None of the subjects had significant restrictive or obstructive ventilatory defect and exhibited tidal intrathoracic EFL in either position during NEP application.

**Table 1:** Anthropometric and functional characteristics of subjects.

	Controls	Snorers	OSAH, mild	OSAH, severe
Subjects (n)	7	14	15	19
Age (yrs)	40 ± 8	49 ± 13	49 ± 10	53 ± 8 *
BMI (kg/m^2^)	24 ± 3	27 ± 4	26 ± 3	29 ± 3 *
FEV1(% pred)	98 ± 10	96 ± 31	103 ± 25	95 ± 26
FVC (% pred)	97 ± 15	96 ± 24	100 ± 24	93 ± 20
FEV1/FVC (%)	88 ± 12	79 ± 11	81 ± 11	86 ± 1
AHI	2 ± 0.4	4 ± 2	18 ± 10	59 ± 16

BMI = body mass index; FEV1 = forced expired volume in the first second; FVC = forced vital capacity; OSAH = obstructive sleep apnea-hypopnea; AHI = apnea-hypopnea index; data are mean ± SD; * p < 0.01 vs Controls.

**Figure 2:**
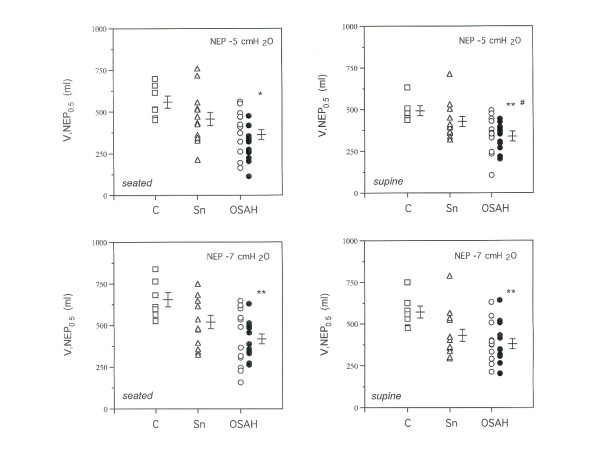
Individual values of V,NEP _0.5_ in seated and supine position at two NEP  levels in patients with obstructive sleep apnea-hypopnea (OSAH) (circles,  white for mild-to-moderate OSAH and black for severe OSAH), snorers (Sn)  (triangles) and controls (C) (squares). *p<0.01 vs C; **p<0.001 vs C; #p<0.05 vs Sn.

The values (mean ± SD) of V,NEP_0.5 _and V,NEP_1_, in both positions and NEP levels, are shown in Table 2. The individual V,NEP_0.5 _data in each group are shown in Fig. 2. Similar values of V,NEP_0.5 _and V,NEP_1 _were obtained in subjects with mild-to-moderate and with severe OSAH in all experimental conditions and were treated as a single group for comparative analysis.

**Table 2:** Values of V,NEP_0.5 _and V,NEP_1 _at two different NEP levels in seated and supine position.

	SEATED		SUPINE	
NEP	-5 cmH_2_O	-7 cmH_2_O	-5 cmH_2_O	-7 cmH_2_O
	V,NEP_0.5_			
	
Controls	559 ± 98	655 ± 113	492 ± 69	571 ± 96
Snorers	457 ± 150	520 ± 147	427 ± 101	430 ± 134
OSAH	363 ± 123*	419 ± 132**	340 ± 88** *#*	379 ± 113**
				
OSAH, m	402 ± 129	428 ± 157	353 ± 103	387 ± 108
OSAH, s	332 ± 112	410 ± 112	329 ± 73	372 ± 119
	V,NEP_1_			
	
Controls	1036 ± 134	1128 ± 158	832 ± 95	920 ± 136
Snorers	864 ± 246	930 ± 274	737 ± 194	755 ± 224
OSAH	708 ± 189*	781 ± 210*	628 ± 136*	669 ± 177*
				
OSAH, m	732 ± 202	766 ± 230	625 ± 159	666 ± 178
OSAH, s	688 ± 182	792 ± 199	629 ± 120	671 ± 180

OSAH = obstructive sleep apnea-hypopnea; m = mild; s = severe; data are mean ± SD; * p < 0.01 vs Controls; ** p < 0.001 vs Controls; *# *p < 0.01 vs Snorers

ΔV,NEP_0.5 _and ΔV,NEP_1 _reflected exactly what was shown by V,NEP_0.5 _and V,NEP_1_with no additional advantage in order to distinguish the different groups. Therefore, we did not consider these time-consuming indices for subsequent analysis.

Within each group V,NEP_0.5 _and V,NEP_1_were significantly higher with NEP -7 cmH_2_O than with NEP -5 cmH_2_O in both positions, and with the same negative pressure higher in the seated position than in the supine one (p < 0.05 for controls, p < 0.01 for snorers and patients with OSAH).

The patients with OSAH consistently exhibited values of V,NEP_0.5 _and V,NEP_1 _much lower than control subjects (p < 0.001), but had values of V,NEP_0.5 _significantly reduced as compared to snorers only with NEP -5 cmH_2_O in the supine position (p < 0.01) (Fig. 2).

**Figure 3:**
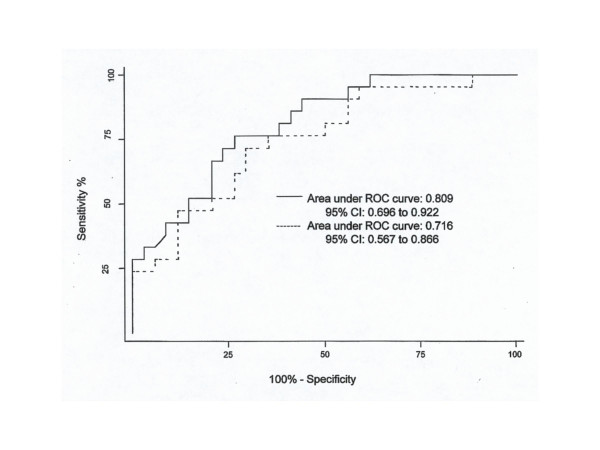
The receiver-operating characteristics (ROC) curves are shown for V,NEP_0.5_  values during NEP (-5 cmH_2_O) both in supine (continuous line) and seated  (dashed line) position in 34 patients with OSAH and 21 subjects without  OSAH. The area under the ROC curves reflects the ability of V,NEP_0.5_ to  distinguish subjects without and with OSAH (AHI>5).

The receiver operating characteristic (ROC) curves performed for V,NEP_0.5 _and V,NEP_1 _in both positions at the two different levels of NEP showed similar areas with the highest value for V,NEP_0.5 _in the supine position using NEP of -5 cmH_2_O (Fig. 3). Under these conditions, the optimal cut-off V,NEP_0.5 _value of 393 ml had a sensitivity of 76% and a specificity of 74% to detect the presence of OSAH with a likelihood ratio for positive results of 2.9. Accordingly, its positive and negative predictive value was 84% and 64%, respectively.

**Figure 4:**
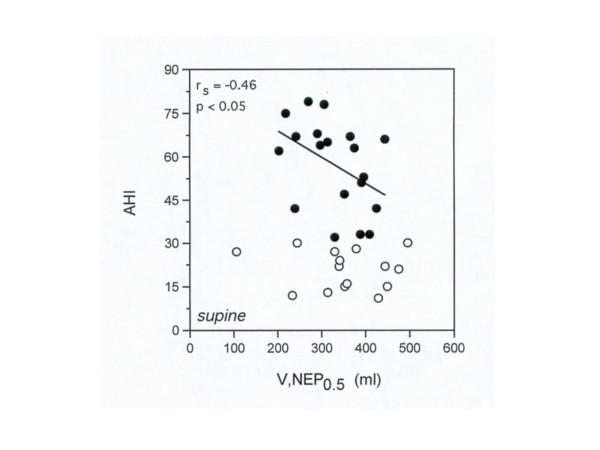
Relationship between AHI and V,NEP_0.5_ in the supine position during NEP (-5  cmH_2_O) in OSAH patients (white circles = mild-to-moderate OSAH; black  circles = severe OSAH). The regression line refers only to severe OSAH  patients.

No significant correlation between V,NEP_0.5 _(in the supine position with NEP level of -5 cmH_2_O) and AHI was observed in patients with OSAH (r_s _= -0.31, r_s_^2 ^= 0.10; 95%IC = -0.59 – 0.04). However, taking into account only the patients with severe OSAH (AHI ≥ 30), a significant inverse correlation was found between V,NEP_0.5 _and AHI (p < 0.05; r_s _= -0.46, r_s_^2 ^= 0.21; 95%IC = -0.76 – -0.01) (Fig. 4).

## Discussion

The present study indicates that during wakefulness OSAH patients when compared to snorers and controls have greater collapsibility of the upper airways which can be easily assessed looking at the early expiratory flow dynamics after NEP application during tidal breathing and properly measured as V,NEP_0.5 _and V,NEP_1_. Such measurements, however, are unable to distinguish on an individual basis apneic from non-apneic snorers because of the overlapping of the V,NEP_0.5 _and V,NEP_1 _values between these groups of subjects. Nevertheless, our results provide support to the idea that a high degree of the upper airway collapsibility promotes OSAH, even if OSAH may seldom occur in subjects with normal upper airway mechanics during wakefulness, suggesting the involvement of other pathogenetic factors.

Although our snorers had similar age, gender and BMI without obvious cranio-facial and ORL anomalies, lateral cephalometry or MRI studies of the pharynx were not performed, so we cannot exclude minor anatomic abnormalities in bony structure or soft tissue around the pharyngeal airway in OSAH patients. Conversely, careful inspection of the maximal and tidal expiratory flow-volume curves allowed us to rule out the presence of intrathoracic expiratory flow limitation during resting tidal breathing in all subjects. Therefore, we are confident that our subjects had no intrathoracic expiratory flow limitation which might have influenced the upper airway-related expiratory flow dynamics when NEP was applied.

The usefulness of the NEP method to assess the upper airway collapsibility was previously tested in 16 awake subjects known to suffer from sleep-disordered breathing [27]. In contrast to snorers, all patients with OSAH (n = 8) showed a substantial portion (>30%) of the expiratory tidal volume throughout the NEP application (-5 cmH_2_O, in the supine position) with lesser expiratory flow than the one recorded during the previous control tidal expiration.

Subsequently, in a group of 19 patients with OSAH when NEP was applied (-5 and -10 cmH_2_O, in the supine position) the expiratory flow was reduced, when compared with the corresponding spontaneous expiratory flow, during a relevant part of the tidal expiration (>20%) in those (n = 13) with a higher mean apnea-hypopnea index (AHI) [28].

In these studies a significant correlation was found between the percentage of the tidal volume during the NEP application with lower expiratory flow than during the spontaneous breathing and oxygen desaturation index (ODI), in the former, and ODI and AHI, in the latter [27,28]. Hence, the NEP method appeared suitable in order to detect an increased pharyngeal collapsibility in patients with OSAH during wakefulness and perhaps able to predict the severity of OSAH.

Recently, using the same criteria, a large cohort of snoring subjects was examined to assess the capacity of the NEP method to screen apneic from non-apneic subjects [29]. In this study a sensitivity of 81.9% and specificity of 69.1% in predicting OSAH was found when the expiratory flow during NEP (-5 cmH_2_O in supine position) was below that of the previous control expiration for ≥27.5% of the tidal volume. In addition, a significant correlation between NEP induced flow analysis and OSAH severity, as assessed by AHI, was found in the supine position using -5 cmH_2_O of NEP with a coefficient value (r_s _= 0.51) similar to the one we obtained in the severe OSAH patients (r_s _= 0.46).

All of these studies, however, are based on the assumption that abnormal upper airway collapsibility is present or can be identified only when the expiratory flow during NEP becomes lower than the control one. Moreover, such finding has been erroneously taken as a marker of expiratory flow limitation. In contrast, an increased pharyngeal collapsibility can also be reflected by a smaller increase of expiratory flow during NEP. We believe that this flow has to be measured whether or not it is higher, lower or initially higher and then lower (or vice versa) than the flow of the previous tidal expiration. Indeed, judging as abnormal (or quantifying the severity of) the upper airway collapsibility only by computing the percentage of the tidal volume where the expiratory flow during NEP application becomes lesser than the one exhibited in the previous expiration is a poorly reliable tool. This is because such measure is too dependent from the preceding control tidal breathing and because the expiratory flow profile is often erratic in the same subject during repeated NEP tests; in addition, several apneic patients do not show such phenomenon constantly [27-29].

In order to overcome these problems, recently Tamisier and coll. investigated a quantitative index corresponding to the ratio of the area under the expiratory flow/volume curves between NEP (-5 and -10 cmH_2_O) and atmospheric pressure for the same tidal volume in awake subjects with sleep disordered breathing (SDB) and control subjects, both in supine and sitting position [30]. They found that this index was significantly different between controls and SDB subjects in all measurements, decreasing with the severity of the SDB. Moreover, in the supine position when -5 cmH_2_O NEP was applied, a given threshold of this index had a positive predictive value of 88.6% and a negative predictive value of 80% to screen subjects with SDB. The Authors concluded that the NEP-related quantitative index may be useful to detect abnormal upper airway collapsibility in awake subjects with SDB. However, some limits of this study are obvious such as the lack of subjects with mild OSAH (AHI <15 and >5) and the age of the controls who were much younger (34 ± 12 yrs) than the patients with OSAH. Furthermore, the application of NEP near end expiratory lung volume tends to elicit reflex activation of genioglossus [22]. This can unpredictably influence the area under the final part of the expiratory flow/volume curve during NEP both in controls and SDB subjects, affecting the quantitative index used to assess the upper airway collapsibility.

In a very recent paper Insalaco and coworkers used the drop of expiratory flow under NEP (ΔV-NEP), expressed as percentage change of peak expiratory flow under NEP, as index of upper airway collapsibility to detect OSAH in patients with sleep disordered breathing. Although this index was a better indicator of OSAH severity when compared to the previous ones, they reported, at best, a determination coefficient equal to 0.32 between the AHI and ΔV-NEP [31], using NEP of -10 cmH_2_O in the supine position. An inherent problem with this approach is that ΔV-NEP does not take into account the duration of the expiratory flow drop under the NEP application, while it is very clear from the flow-time tracings given by the same Authors that this transient may last very differently with the same percentage value of reduction.

By time-integration of the expiratory flow in the first 0.5 and 1 sec after the application of a given level of NEP one can easily calculate the expiratory volume exhaled in a preset time in a given body position during wakefulness and use this parameter as an index of the mechanical properties of the upper airways at the onset of expiration when the genioglossus does not appear reflexively activated [22]. Therefore, the novelty of this study is the utilization of a method which, still adopting the NEP technique, is more reliable to assess and measure the upper airway collapsibility because it is quantitative, and it is not influenced by the flow of the preceding tidal expiration and by the effect of neuromuscular factors.

V,NEP_0.5 _and V,NEP_1 _values were reduced in the supine position at each level of applied NEP in all groups, likely to reflect a posture-related increase in the upper airway resistance [18,23,32,33]. Therefore, V,NEP_0.5 _and V,NEP_1 _measurements appear to be influenced by the baseline expiratory upper airway resistance which has been shown to be higher in OSAH patients, probably because of minimal structural abnormalities (abnormal hyoid bone position and increase in soft pharyngeal tissues) [20] and related shape changes. It is conceivable that lower V,NEP_0.5 _and V,NEP_1 _found in our OSAH patients may be partly due to reduced baseline upper airway caliber which, on the other hand, is expected to increase the pharyngeal compliance and finally the upper airway collapsibility in these subjects [21]. However, the expired volume in the first 0.5 or 1 sec was lower during NEP than during the previous control expiration in either position in about 15–18% of our apneic snorers. This never occurred in snorers and controls. This fact strongly suggests that in OSAH patients a brisk narrowing of upper airways is elicited by the sudden NEP application, the magnitude of which is largely depending on the pharyngeal collapsibility under the prevailing circumstances and substantially reflected by the V,NEP_0.5 _or V,NEP_1 _values. In line with this reasoning, the early expiratory flow during NEP was often below the isovolume spontaneous expiratory flow, particularly in OSAH patients (see Fig. 1), as also shown in previous studies [22,27,28].

Under these experimental conditions, V,NEP_0.5 _was significantly lower in apneic than in non-apneic snorers when measured in the supine position utilizing the smallest level of NEP (i.e.: -5 cmH_2_O in our study). Thus, the lower the value of V,NEP_0.5 _(or V,NEP_1_), the higher the possibility for snoring people to have OSAH. Indeed, according to this method an increased pharyngeal collapsibility even during wakefulness affects the vast majority of snorers who have OSAH. This information is obtained in a rapid, simple and non-invasive way without cooperation of the subjects who can be studied when awake, repeatedly and in different body position. In this respect it has to be stressed that it is not necessary to control with regards to baseline spontaneous tidal volumes and flows. Indeed, ΔV,NEP_0.5 _and ΔV,NEP_1 _did not perform differently or better to distinguish between snorers and OSAH patients than V,NEP_0.5 _and V,NEP_1_.

Unfortunately, the ability to differentiate snorers with or without OSAH was not sufficient, at least within our capabilities, to recommend this technique and related parameters as a reliable diagnostic tool to obviate sleep studies or even to select subjects for polysomnographic evaluation. Lower levels of NEP (i.e.: -2 or -3 cmH_2_O), however, might be more useful for this purpose and deserve to be tested in the future.

Three further comments need to be made. Firstly, generally a high collapsibility of the upper airways does not seem sufficient to cause OSAH since several snorers without OSAH exhibited similarly reduced values of V,NEP_0.5 _or V,NEP_1_. Secondly, other factors must influence the severity of OSAH, as assessed by ODI and AHI, because no different values of V,NEP_0.5 _(or V,NEP_1_) were found in any position or with different levels of NEP between mild-to-moderate and severe OSAH patients. Thirdly, some OSAH patients have surprisingly high values of V,NEP_0.5 _(or V,NEP_1_) comparable to those of the controls, showing a normal upper airway collapsibility during wakefulness, and thus suggesting different state-related factors leading to OSAH or a site of upper airway obstruction during sleep only at naso-pharyngeal level which cannot be directly assessed with this technique.

Finally, contrary to the opinion of the other Authors who used the NEP technique to detect OSAH patients during wakefulness [27-30], we have to stress that, although the results obtained with V,NEP_0.5 _were similar or even better than the previous ones [27-30], whatever NEP-related parameter is adopted, presently this tool is not sufficiently capable of revealing OSAH on an individual basis for clinical purpose.

In conclusion, the NEP technique when properly used is potentially useful to study upper airway collapsibility in patients with OSAH during wakefulness in order to better understand its main mechanisms, to assess in the long term the effects of various interventions, and possibly for selecting non-apneic snorers to follow up. On the other hand, it cannot be recommended for routine OSAH screening in awake snorers who should subsequently be subjected to sleep studies.

## Abbreviations

OSAH = obstructive sleep apnea-hypopnea; AHI = apnea-hypopnea index; NEP = negative expiratory pressure; SDB = sleep disordered breathing; BMI = body mass index; V,NEP_0.5 _and V,NEP_1 _= exhaled volume in the first 0.5 and 1 sec. after application of NEP; ROC = receiver operating characteristic (curve)
